# Pulmonary Artery Mass Caused by *Aspergillus fumigatus* Infection After Heart Transplantation: A Case Report

**DOI:** 10.3390/idr18040085

**Published:** 2026-08-10

**Authors:** Gang Liu, Zhongkai Liao, Jie Huang, Zhiyuan Zhu, Mingzhu Liu, Sheng Liu, Ru Liu

**Affiliations:** 1 Department of Cardiovascular Surgery, TEDA International Cardiovascular Hospital, Tianjin University, Chinese Academy of Medical Sciences, Peking Union Medical College, No. 61, 3rd Avenue, TEDA, Tianjin 300457, China; liugang1026@foxmail.com; 2 Department of Adult Cardiac Surgery, Fuwai Hospital, National Center for Cardiovascular Diseases, Chinese Academy of Medical Sciences, Peking Union Medical College, No. 167 North Lishi Road, Xicheng District, Beijing 100037, China; lion1972200@aliyun.com (Z.L.); huangjie@fuwai.com (J.H.); zhuzhiyuanstudy@163.com (Z.Z.); liusheng@fuwai.com (S.L.); 3 Department of Heart Failure and Transplantation, Fuwai Hospital, National Center for Cardiovascular Diseases, Chinese Academy of Medical Sciences, Peking Union Medical College, No. 167 North Lishi Road, Xicheng District, Beijing 100037, China; 4 Department of Cardiovascular Surgery, The First Affiliated Hospital with Nanjing Medical University, 300 Guangzhou Road, Gulou District, Nanjing 210029, China; liumingzhu1996@126.com; 5 Department of Cardiology, Fuwai Hospital, National Center for Cardiovascular Diseases, Chinese Academy of Medical Sciences, Peking Union Medical College, No. 167 North Lishi Road, Xicheng District, Beijing 100037, China

**Keywords:** case report, heart transplantation, invasive fungal infection, *Aspergillus fumigatus*, *Streptococcus mitis*, next-generation sequencing, panel reactive antibody

## Abstract

**Background**: Invasive aspergillosis (IA) is a severe complication after solid organ transplantation, especially in patients receiving long-term immunosuppressive therapy. Pulmonary artery involvement caused by *Aspergillus fumigatus* is extremely rare and may mimic pulmonary artery thrombosis or malignancy, resulting in diagnostic challenges. **Case Presentation**: A 61-year-old male developed fatigue, intermittent fever, cough, and chest discomfort seven months after orthotopic heart transplantation. Computed tomography pulmonary angiography revealed a mass-like filling defect involving the main pulmonary artery and left pulmonary artery, which was initially suspected to represent pulmonary artery thrombosis or tumor. Whole-blood next-generation sequencing identified *Aspergillus fumigatus* and *Streptococcus mitis*. The patient was treated with voriconazole, anticoagulation therapy, and adjustment of immunosuppressive therapy. Follow-up imaging demonstrated progressive regression of pulmonary artery lesions and disappearance of pulmonary nodules without evidence of graft rejection. **Conclusions**: Pulmonary artery aspergillosis should be considered in heart transplant recipients presenting with pulmonary artery-occupying lesions, particularly when imaging findings are atypical. Early pathogen identification combined with appropriate antifungal therapy, individualized immunosuppression adjustment, and careful monitoring may improve clinical outcomes.

## 1. Introduction

Invasive fungal infections (IFIs) remain among the most serious infectious complications after solid organ transplantation (SOT), particularly because of their high morbidity and mortality in immunocompromised recipients. Among these infections, invasive aspergillosis (IA) is one of the most important opportunistic fungal diseases in transplant recipients [1,2]. Heart transplant recipients are particularly vulnerable due to long-term exposure to immunosuppressive agents, impaired cellular immunity, repeated hospitalization, and other factors affecting host defense mechanisms.

The development of IA after transplantation is associated with multiple risk factors, including prolonged corticosteroid therapy, intensive immunosuppressive regimens, lymphopenia, cytomegalovirus infection, renal dysfunction, prolonged intensive care unit stay, and exposure to broad-spectrum antibiotics [3,4]. Although antifungal prophylaxis and improved diagnostic strategies have reduced the incidence of IA, delayed diagnosis remains a major challenge because clinical manifestations are often nonspecific and may overlap with other conditions such as bacterial infection, malignancy, and thromboembolic disease.

The lung is the most common site of invasive aspergillosis. However, Aspergillus infection may also disseminate through vascular invasion and involve extrapulmonary organs, including the central nervous system, heart, and liver [1,5]. Hepatic Aspergillus abscesses and cerebral aspergillosis are uncommon but severe manifestations, often associated with poor outcomes. Vascular invasion by Aspergillus may lead to unusual radiological presentations, including vascular occlusion, aneurysm formation, or mass-like lesions that mimic thrombotic or malignant diseases.

Pulmonary artery involvement caused by Aspergillus is extremely rare, especially after heart transplantation [6]. Such lesions may be difficult to distinguish from pulmonary embolism or pulmonary artery sarcoma based solely on imaging findings. Accurate pathogen identification and timely initiation of targeted antifungal therapy are therefore essential. In this report, we describe a rare case of *Aspergillus fumigatus* infection presenting as a pulmonary artery mass after heart transplantation, initially mimicking pulmonary artery thrombosis. This case highlights the diagnostic challenges and therapeutic considerations associated with atypical vascular manifestations of invasive aspergillosis in transplant recipients.

## 2. Case Presentation

### 2.1. Patient Information

A 61-year-old man with end-stage heart failure secondary to arrhythmogenic left ventricular cardiomyopathy underwent orthotopic heart transplantation. The postoperative immunosuppressive regimen consisted of prednisone, mycophenolate mofetil (MMF), and cyclosporine because of rapid tacrolimus metabolism. Before the onset of infection, the cyclosporine trough concentration was maintained at 200–250 ng/mL, while the CD4^+^ T-cell count remained persistently below 200 cells/μL, indicating sustained cellular immunosuppression.

### 2.2. Clinical Presentation

Seven months after heart transplantation, the patient presented with progressive fatigue, intermittent fever, productive cough, and pleuritic chest pain for approximately two weeks. Initial evaluation at a local hospital revealed elevated inflammatory markers and a mildly increased serum (1→3)-β-D-glucan (G test). Computed tomography pulmonary angiography (CTPA) demonstrated a mass-like filling defect involving the main pulmonary artery and the left pulmonary artery, accompanied by multiple bilateral pulmonary nodules. The initial differential diagnosis included pulmonary artery thrombosis and pulmonary artery sarcoma. Empirical antimicrobial therapy with cefotaxime/sulbactam together with anticoagulation using low-molecular-weight heparin was initiated; however, the patient’s symptoms showed no significant improvement. He was therefore referred to our center for further diagnostic evaluation and treatment.

### 2.3. Diagnostic Assessment

At admission, laboratory investigations demonstrated no marked leukocytosis (white blood cell count, 6.80 × 10^9^/L), whereas inflammatory markers, including C-reactive protein (CRP), were elevated. Serum (1→3)-β-D-glucan (G test) was mildly increased, with an initial value of 90.5 pg/mL and a peak value of 174.1 pg/mL during hospitalization. Serum galactomannan (GM) was monitored serially during treatment. D-dimer was mildly elevated (0.34 μg/mL). Panel reactive antibody (PRA), a marker of anti-human leukocyte antigen (HLA) sensitization and immunological rejection risk, remained negative throughout hospitalization. Lymphocyte subset analysis demonstrated persistent CD4^+^ T-cell lymphopenia (<200 cells/μL), which gradually recovered following reduction of immunosuppressive therapy.

Whole-blood metagenomic next-generation sequencing (mNGS), performed by a certified third-party clinical laboratory, identified *Aspergillus fumigatus* (relative abundance, 50.7%) and *Streptococcus mitis* (relative abundance, 10.04%). The microbiological findings were interpreted in conjunction with the patient’s clinical manifestations, laboratory results, and imaging findings, supporting the diagnosis of invasive pulmonary aspergillosis with pulmonary artery involvement complicated by *Streptococcus mitis* co-infection.

Transthoracic echocardiography demonstrated preserved cardiac graft function without significant valvular abnormalities. However, repeat examination revealed a hypoechoic mass extending from the main pulmonary artery to the left pulmonary artery. No evidence of deep venous thrombosis was identified on lower-extremity venous ultrasonography. The overall clinical course is summarized in Table 1, and key admission laboratory findings are provided in Table 2.

**Table 1:** Timeline of Clinical Events.

Time	Clinical Events
Heart transplantation	Orthotopic heart transplantation; triple immunosuppressive therapy initiated
7 months after transplantation	Fatigue, intermittent fever, productive cough, and chest pain
Initial local evaluation	CTPA revealed a pulmonary artery mass with bilateral pulmonary nodules (Figure 1A,B)
Hospital admission	Multidisciplinary consultation and further diagnostic work-up
Day 3	Whole-blood mNGS detected *Aspergillus fumigatus* and *Streptococcus* mitis
Day 5	Voriconazole therapy initiated; immunosuppression reduced
3 weeks after treatment	CTPA demonstrated reduction of pulmonary artery lesions (Figure 1C,D)
7 months after discharge	Pulmonary nodules resolved and pulmonary artery lesion markedly decreased (Figure 1E,F)

**Table 2:** Summary of Laboratory Findings at Admission.

Parameter	Result	Reference Range
White blood cell count	6.80 × 10^9^/L	3.5–9.5 × 10^9^/L
Neutrophils	70.7%	40–75%
C-reactive protein	Elevated	<8 mg/L
D-dimer	0.34 μg/mL	<0.5 μg/mL
β-D-glucan (G test)	90.5 pg/mL (peak 174.1 pg/mL)	<95 pg/mL
Galactomannan (GM)	0.56 μg/L (peak 1.06 μg/L)	<0.25 μg/L
CD4^+^ T-cell count	<200 cells/μL	>500 cells/μL
Panel reactive antibody (PRA)	Negative	Negative
Whole-blood mNGS	*A. fumigatus* (50.7%), *S. mitis* (10.04%)	Negative

**Figure 1:**
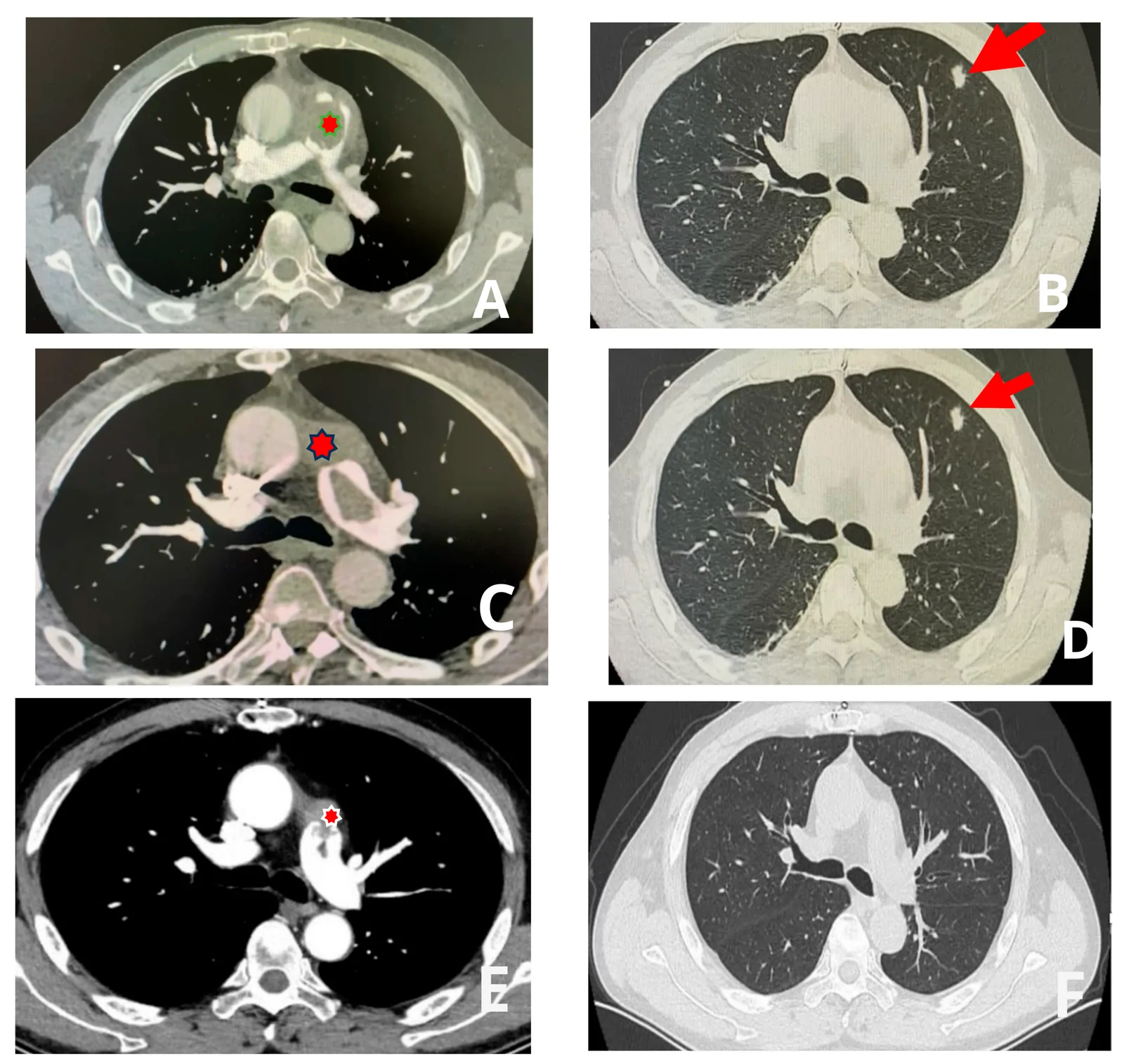
CTPA (initial local evaluation). (**A**) A massive filling defect in the main pulmonary artery and left pulmonary artery trunk; (**B**) multiple nodules in both lungs, more prominent in the left lung. CTPA three weeks after drug treatment. (**C**,**D**) All lesions were reduced. CTPA seven months later. (**E**) The lesion in the pulmonary artery was significantly reduced; (**F**) the intrapulmonary lesions had nearly disappeared. The red asterisk indicates the pulmonary artery lesion, and the red arrow indicates pulmonary nodules.

**Figure 2:**
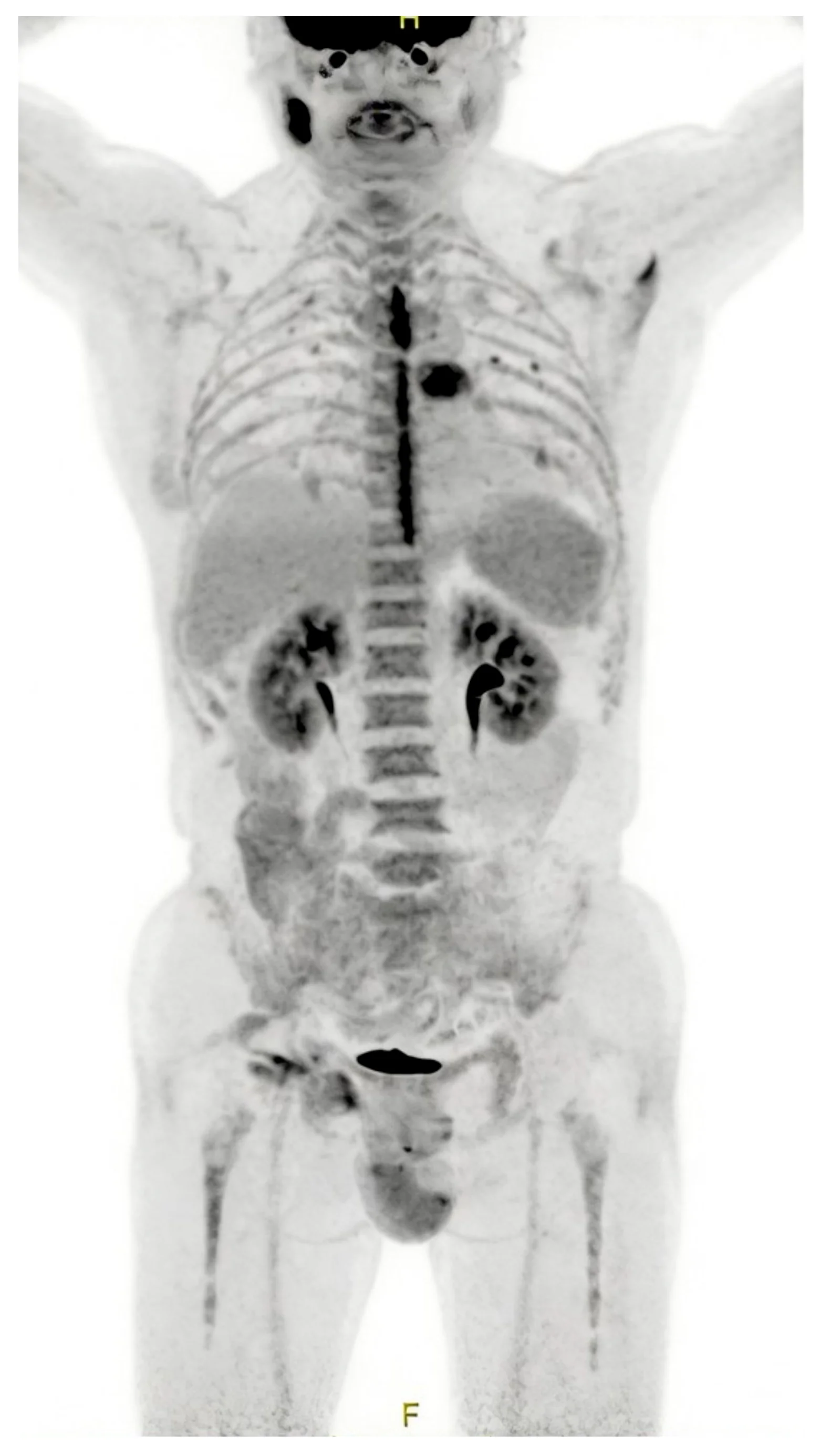
PET/CT. A high-density shadow in the proximal pulmonary artery trunk (3.2 cm × 3.4 cm), SUVmax 9.1, and multiple solid nodular shadows in both lungs, SUVmax 2.1–3.7, were considered infectious lesions. The letter F denotes fluorine-18 in 18F-FDG.

Computed tomography pulmonary angiography (CTPA) demonstrated a 32 × 28 mm mass-like filling defect involving the main pulmonary artery and extending into the left pulmonary artery, accompanied by multiple bilateral pulmonary nodules. These findings raised concern for pulmonary artery thrombosis or pulmonary artery sarcoma. To further characterize the lesion, ^18F-fluorodeoxyglucose positron emission tomography/computed tomography (^18F-FDG PET/CT) was performed after at least 6 h of fasting. The patient’s blood glucose level before intravenous administration of 250 MBq of ^18F-FDG was 5.6 mmol/L. PET/CT demonstrated increased metabolic activity within the pulmonary artery lesion (SUV_max 9.1) and multiple pulmonary nodules (SUV_max 2.1–3.7), findings that favored an infectious rather than malignant process (Figure 2).

### 2.4. Therapeutic Intervention

Following multidisciplinary consultation involving specialists in cardiac surgery, cardiology, infectious diseases, radiology, nuclear medicine, and pulmonary medicine, invasive pulmonary aspergillosis with pulmonary artery involvement was considered the most likely diagnosis. Although pulmonary artery thrombosis and pulmonary artery sarcoma could not be completely excluded at the initial stage, the patient’s immunocompromised status, radiological findings, positive fungal biomarkers, and mNGS results strongly supported an infectious etiology. Consequently, a comprehensive treatment strategy consisting of targeted antifungal therapy, short-course antibacterial therapy, anticoagulation, and individualized adjustment of immunosuppressive therapy was initiated.

Voriconazole was selected as first-line antifungal therapy and administered orally at a dose of 200 mg twice daily. Therapeutic drug monitoring was performed regularly, and the trough concentration was maintained between 2 and 6 mg/L throughout treatment. Considering the concomitant detection of *Streptococcus mitis* by mNGS and the possibility of bacterial co-infection, empirical antibacterial therapy with moxifloxacin was administered for one week and discontinued after clinical improvement and normalization of inflammatory markers.

Because pulmonary artery thrombosis could not be definitively excluded at presentation, anticoagulation was initiated with low-molecular-weight heparin (LMWH). After the patient’s clinical condition stabilized, anticoagulation was transitioned to oral warfarin, with the international normalized ratio (INR) maintained between 2.0 and 2.5 during follow-up.

Given that excessive immunosuppression was considered an important predisposing factor for invasive aspergillosis, the immunosuppressive regimen was carefully modified while maintaining adequate graft protection. Mycophenolate mofetil was discontinued because of the severe opportunistic fungal infection, whereas low-dose prednisone was continued. The cyclosporine dose was gradually reduced to achieve a target trough concentration of 100–200 ng/mL. Throughout treatment, graft function was closely monitored by serial echocardiography together with assessment of panel reactive antibody (PRA), lymphocyte subsets, and cyclosporine trough concentrations. No clinical or laboratory evidence of acute graft rejection was observed during hospitalization or subsequent follow-up. The patient tolerated the combined therapeutic regimen well, and no significant drug-related adverse events, including hepatotoxicity, nephrotoxicity, or severe hematological toxicity, were observed.

### 2.5. Follow-Up and Outcomes

The patient became afebrile within 48 h after initiation of antifungal therapy, and respiratory symptoms gradually resolved during hospitalization. Serum inflammatory markers, including C-reactive protein, progressively normalized. Serial monitoring demonstrated a gradual decline in serum (1→3)-β-D-glucan (G test) and galactomannan (GM) levels, indicating a favorable response to antifungal therapy. Following reduction of immunosuppressive intensity, the CD4^+^ T-cell count gradually recovered from <200 cells/μL to approximately 500–600 cells/μL.

Follow-up CTPA performed three weeks after treatment initiation demonstrated partial regression of the pulmonary artery filling defect and bilateral pulmonary nodules. At the 7-month follow-up, repeat CTPA showed near-complete resolution of the pulmonary nodules and marked reduction of the pulmonary artery lesion. These radiological findings were consistent with sustained clinical improvement.

Voriconazole therapy was continued for a total of seven months with regular therapeutic drug monitoring. The patient tolerated the treatment well, and no clinically significant adverse drug reactions, including hepatotoxicity, nephrotoxicity, or hematological toxicity, were observed. During follow-up, serial echocardiography demonstrated preserved cardiac graft function, and no evidence of acute rejection was identified based on clinical evaluation, panel reactive antibody monitoring, or routine cardiac assessment. At the most recent follow-up visit, the patient had returned to normal daily activities with a satisfactory quality of life. This case report was prepared in accordance with the CARE guidelines (Table S1).

## 3. Discussion

Invasive aspergillosis (IA) remains one of the most severe opportunistic fungal infections after solid organ transplantation (SOT), particularly among patients receiving prolonged immunosuppressive therapy [7]. In the present case, *Aspergillus fumigatus* infection developed after heart transplantation and manifested as a pulmonary artery mass mimicking pulmonary artery thrombosis, representing an extremely uncommon vascular presentation of invasive fungal infection [8,9,10]. This atypical manifestation created substantial diagnostic challenges and highlights the importance of considering fungal infection in transplant recipients with unexplained pulmonary vascular lesions.

Heart transplant recipients are particularly susceptible to invasive fungal infections because of impaired cellular immunity caused by long-term immunosuppressive therapy [3]. Previously reported risk factors include high-intensity immunosuppression, corticosteroid exposure, lymphopenia, cytomegalovirus infection, prolonged hospitalization, renal dysfunction, and broad-spectrum antibiotic exposure [4,11,12]. The incidence of invasive pulmonary aspergillosis (IPA) after heart transplantation remains relatively low but is associated with considerable mortality, with reported mortality rates exceeding 30% in many cohorts [7]. Early diagnosis and timely initiation of effective antifungal therapy are therefore critical for improving outcomes [13,14,15,16,17,18]. International studies such as Yetmar et al. [3] showed that when the trough concentration of cyclosporine is maintained at >200 ng/mL for a long time or the trough concentration of tacrolimus is >10 ng/mL, the risk of IFI in heart transplant recipients increases by 3.5 times, and the risk is further increased when combined with MMF and high-dose glucocorticoids. CD4^+^T lymphocyte count is the most sensitive immune function index for predicting fungal infection in post-transplant recipients, and CD4^+^T lymphocyte count <200/μL is considered the high-risk threshold for IFI [3,7]. Singh et al. [7] found that in heart transplant recipients with CD4^+^T lymphocyte count <200/μL for more than 3 months, the incidence of IPA was 21.3%, while the incidence was only 2.8% in those with count >500/μL. In our patient, persistent CD4^+^ T-cell lymphopenia and sustained immunosuppressive exposure represented important predisposing factors for invasive fungal infection.

The clinical manifestations and outcomes of IA vary according to the involved organs and extent of dissemination. Pulmonary infection is the most common manifestation and may present with nodules, consolidation, cavitary lesions, or angioinvasive changes. However, Aspergillus can disseminate beyond the lungs and cause severe extrapulmonary disease. Central nervous system aspergillosis is one of the most devastating forms of IA, with high mortality despite antifungal therapy because of delayed diagnosis and limited penetration of antifungal agents into infected tissues. Aspergillus hepatic abscess is another rare but serious manifestation, predominantly occurring in immunocompromised patients, and is often associated with nonspecific clinical symptoms and diagnostic difficulty. Therefore, clinicians should maintain a high suspicion of IA when transplant recipients present with unusual lesions involving different organs [5].

The diagnosis of IA remains challenging because clinical manifestations and imaging findings are frequently nonspecific. In this case, the pulmonary artery lesion initially suggested pulmonary artery thrombosis or pulmonary artery sarcoma based on CTPA findings. Conventional diagnostic approaches, including fungal culture, antigen detection, and imaging studies, remain essential but may have limited sensitivity, particularly in patients with atypical presentations. Metagenomic next-generation sequencing (mNGS) has emerged as a valuable complementary diagnostic technique because it enables rapid and unbiased detection of pathogens directly from clinical specimens [6,19]. In this patient, whole-blood mNGS identified A. fumigatus and supported early initiation of targeted therapy. However, mNGS results should always be interpreted in combination with clinical manifestations, imaging findings, and other microbiological evidence, as detection of microbial nucleic acids does not necessarily indicate active infection.

In addition to pathogen identification, antifungal susceptibility testing and minimum inhibitory concentration (MIC) determination are essential components of individualized antifungal management. Increasing reports of azole-resistant A. fumigatus infections have raised concerns regarding treatment failure, particularly in patients with resistance-associated genetic alterations such as CYP51A mutations. Elevated MIC values may indicate reduced susceptibility to specific antifungal agents and should guide therapeutic decisions, including modification of antifungal regimens or consideration of combination therapy. Although antifungal susceptibility testing could not be performed in this case because no viable fungal isolate was obtained, MIC testing should be considered whenever possible in invasive fungal infections, especially in patients with prolonged infection, prior azole exposure, or poor clinical response.

Treatment of IA after heart transplantation requires a multidisciplinary approach that balances infection control and preservation of graft function [2]. Voriconazole remains a recommended first-line therapy for IPA, and therapeutic drug monitoring is particularly important because of considerable pharmacokinetic variability and interactions with immunosuppressive agents. In the present case, prolonged voriconazole therapy combined with anticoagulation and individualized reduction of immunosuppressive intensity resulted in progressive radiological improvement without evidence of graft rejection. The reduction of immunosuppression requires careful balancing, as excessive reduction may increase rejection risk, whereas persistent immunosuppression may compromise infection control. Therefore, individualized adjustment guided by clinical response, immune monitoring, and graft function assessment is essential.

Pulmonary artery involvement caused by Aspergillus is extremely rare, and only limited cases have been reported. Previous reports suggest that conservative medical management with antifungal therapy may achieve favorable outcomes in selected patients when surgical intervention carries high risk [20,21]. In our patient, the combination of early pathogen identification, targeted antifungal therapy, anticoagulation, and careful immunosuppressive adjustment resulted in significant lesion regression and long-term clinical stability. This case emphasizes that Aspergillus infection should be included in the differential diagnosis of pulmonary artery masses in transplant recipients, even when imaging findings initially suggest thromboembolic disease.

## 4. Conclusions

Application of immunosuppressants by heart recipients is a risk factor for fungal infections, especially in those whose CD4^+^T lymphocytes are below 200/μL. The intensity of immunosuppression should be adjusted individually to reduce infection risk. NGS has high diagnostic value for the diagnosis of fungal infections, especially in patients with rare imaging presentations. Early identification of pathogens, targeted anti-pathogenic treatment with an adequate course, and simultaneous monitoring of rejection are the key points to improve the prognosis of patients with fungal infections after heart transplantation.

## Data Availability

The data presented in this case report are available from the corresponding author upon reasonable request. The data are not publicly available due to patient privacy.

## Supplementary Materials

The following supporting information can be downloaded at https://www.mdpi.com/article/10.3390/idr18040085/s1, Table S1: CARE Checklist.
