# Supplementary material for: Pulmonary Artery Mass Caused by Aspergillus fumigatus Infection After Heart Transplantation: A Case Report
Source: Infect Dis Rep. 2026 Aug 10;18(4):85. doi: 10.3390/idr18040085 (PMC13511923; doi:10.3390/idr18040085)
Supplement: Supplementary file 1 [file idr-18-00085-s001.zip › idr-4398165-supplementary.pdf]

CARE Checklist

Pulmonary Artery Mass Caused by Aspergillus fumigatus Infection After Heart Transplantation: A Case Report

Item	CARE checklist item	Recommendation	Reported in manuscript
1	Title	The words "case report" should appear in the title together with the phenomenon of greatest interest.	Page 1, title
2	Key words	Provide 2–5 key words identifying the diagnoses or interventions, including "case report."	Page 1, Keywords
3a	Abstract—Introduction	State what is unique about the case and what it adds to the literature.	Page 1, Abstract—Background
3b	Abstract—Main symptoms and/or findings	Summarize the patient's main symptoms and important clinical findings.	Page 1, Abstract—Case Presentation
3c	Abstract—Main diagnoses, interventions, and outcomes	Summarize the principal diagnosis, therapeutic interventions, and outcomes.	Page 1, Abstract—Case Presentation and Conclusions
3d	Abstract—Conclusion	State the principal lesson or take-away message.	Page 1, Abstract—Conclusions
4	Introduction	Provide a brief background summary explaining why the case is noteworthy, with relevant literature.	Pages 1–2, Introduction
5a	Patient information—Demographics	De-identified demographic and other relevant patient information.	Page 2, Section 2.1
5b	Patient information—Symptoms and concerns	Describe the patient's main symptoms and concerns.	Pages 2–3, Section 2.2
5c	Patient information—Medical, family, and psychosocial history	Include relevant medical, family, and psychosocial history, including genetic information when appropriate.	Page 2, Section 2.1; family and psychosocial history not clinically relevant/not reported
5d	Patient information—Past interventions	Describe relevant previous interventions and their outcomes.	Pages 2–3, Sections 2.1–2.2
6	Clinical findings	Describe relevant physical examination and other important clinical findings.	Pages 2–5, Sections 2.2–2.3; physical examination findings were not specifically reported
7	Timeline	Present important events in chronological sequence, preferably in a table or figure.	Page 3, Table 1
8a	Diagnostic assessment—Methods	Describe diagnostic methods, including physical examination, laboratory testing, imaging, surveys, and other investigations.	Pages 3–5, Section 2.3; Table 2; Figures 1–2
8b	Diagnostic assessment—Challenges	Describe diagnostic challenges, including access, financial, cultural, or other barriers when applicable.	Pages 2–3 and 6–8, differential diagnosis and diagnostic difficulty; no access/financial/cultural barriers reported
8c	Diagnostic assessment—Diagnosis and differential diagnosis	State the diagnosis and relevant differential diagnoses.	Pages 2–5, Sections 2.2–2.4
8d	Diagnostic assessment—Prognostic characteristics	Include prognostic characteristics or staging where applicable.	Pages 3–4 and 6–8, immune status, CD4+ T-cell lymphopenia, biomarkers, and imaging burden
9a	Therapeutic intervention—Types	Describe the types of therapeutic intervention used.	Pages 5–6, Section 2.4
9b	Therapeutic intervention—Administration	Describe dose, strength, duration, route, and other administration details.	Pages 5–6, Sections 2.4–2.5
9c	Therapeutic intervention—Changes	Describe changes in treatment and provide the rationale.	Pages 5–6, Section 2.4
10a	Follow-up and outcomes—Clinician/patient assessed outcomes	Report clinician- and patient-assessed outcomes when available.	Page 6, Section 2.5

Item	CARE checklist item	Recommendation	Reported in manuscript
10b	Follow-up and outcomes—Follow-up tests	Report important follow-up diagnostic and other test results.	Page 6, Section 2.5; Figure 1
10c	Follow-up and outcomes—Adherence and tolerability	Describe intervention adherence and tolerability, including how these were assessed.	Pages 5–6, Sections 2.4–2.5
10d	Follow-up and outcomes—Adverse and unanticipated events	Describe adverse and unanticipated events.	Page 6, no clinically significant drug-related adverse events observed
11a	Discussion—Strengths and limitations	Discuss the strengths and limitations of the case report.	Pages 7–8, Discussion; limitation regarding unavailable fungal isolate/MIC testing
11b	Discussion—Relevant literature	Discuss the relevant medical literature.	Pages 6–8, Discussion
11c	Discussion—Rationale for conclusions	Provide the scientific rationale for the conclusions.	Pages 6–8, Discussion and Conclusions
11d	Discussion—Take-away lesson	State the primary take-away lesson in one paragraph.	Page 8, final Discussion paragraph and Conclusions
12	Patient perspective	Include the patient’s perspective or experience when appropriate.	Not reported
13	Informed consent	Confirm that informed consent for publication was obtained.	Page 9, Informed Consent Statement

Note: The patient perspective was not included in the manuscript. This CARE item may be marked “Not reported” unless the authors elect to add a brief patient-perspective statement.
